# How Long Is Long COVID? Evaluation of Long-Term Health Status in Individuals Discharged from a Specialist Community Long COVID Service

**DOI:** 10.3390/jcm13195817

**Published:** 2024-09-29

**Authors:** Rochelle Bodey, Jennifer Grimaldi, Hannah Tait, Belinda Godfrey, Sharon Witton, Jenna Shardha, Rachel Tarrant, Manoj Sivan

**Affiliations:** 1 Leeds Long COVID Community Rehabilitation Service, Leeds Community Healthcare NHS Trust, Leeds LS11 0DL, UKhannah.tait6@nhs.net (H.T.);; 2 National Demonstration Centre of Rehabilitation Medicine, Leeds Teaching Hospitals NHS Trust, Leeds LS7 4SA, UK; 3 Academic Department of Rehabilitation Medicine, Leeds Institute of Rheumatic and Musculoskeletal Medicine, University of Leeds, Martin Wing, Leeds General Infirmary, Leeds LS1 3EX, UK

**Keywords:** COVID-19, post-COVID-19 condition, post-COVID-19 syndrome, post-acute infection syndrome (PAIS)

## Abstract

**Background:** Post COVID-19 syndrome or long COVID (LC) is a novel fluctuating condition with a protracted course in some patients. Specialist LC services have been operational in the UK since 2020 and deal with a high caseload of patients. **Aims:** To evaluate long-term outcomes in patients discharged from a community-based LC specialist service. **Methods:** A service evaluation study that included patients who were well engaged in the services [completed the standard Patient Reported Outcome Measures (PROMs) and received intervention from clinician(s)] and had been discharged for at least 3 months from the service. They consented to the study and completed standard PROMs: COVID-19 Yorkshire Rehabilitation Scale (C19-YRS), EQ-5D-5L and National Institute for Health and Care Excellence (NICE) criteria for myalgia encephalomyelitis/chronic fatigue syndrome (ME/CFS). **Results:** Out of 460 patients contacted, 112 (average of 37.6 months since infection and 9.8 months post-discharge) completed the PROMs. Of these, 90.2% patients continued to experience LC symptoms and disability and had not returned to their pre-COVID-19 health status. The average EQ-5D-5L index score was 0.53 (SD 0.29), highlighting a significant disability and that LC had become a long-term condition (LTC) in the majority of patients who responded to the survey. Of these patients, 43% met the criteria for suspected ME/CFS. **Conclusions:** A proportion of LC patients develop persistent long COVID (PLC) consistent with a LTC and had a significant overlap with ME/CFS.

## 1. Introduction

Post-COVID-19 syndrome, or long COVID (LC) is defined as the continuation or development of new symptoms 3 months after the initial SARS-CoV-2 infection, with these symptoms lasting at least 2 months with no other explanation [1]. It is estimated that there are approximately 1.9 million people in England experiencing symptoms following COVID-19 in the UK, with 1.1 million having experienced these symptoms for over 12 months and 762,00 (41%) for at least 2 years (persistent LC) [2].

Patients experience debilitating and wide-ranging symptoms across multiple organ systems [3,4], with symptoms such as fatigue, ‘brain fog’, pain and shortness of breath being reported for months after infection [5]. Disability rates are high within this population with >20% of people reporting that their symptoms limit their ability to perform their normal activities ‘a lot’ [2]. Symptom burden and disability in LC have been found to be worse than those reported in the literature for diabetes mellitus, COPD, heart failure and multiple sclerosis [6].

The prevalence of self-reported LC in the UK is greatest amongst people aged 35–69 [2], many reporting the symptoms having an impact on their ability to work and remain in employment. In a survey by the Trade Union Congress in March 2023, one in seven respondents (14%) had lost their job because of reasons connected to LC [7] and one in four employers now include LC among their main causes of long-term sickness absence [8].

With symptoms lasting for years [5,6,9] there is emerging evidence that LC should now be considered for some patients as a long-term condition (LTC). One UK evaluation showed no improvement in EQ-5D-5L at 6-month follow-up and concluded that for most patients in this evaluation, LC had evidently become a long-term condition, causing disability and significant deterioration of their overall health status even 18 months post infection [6]. Persistent symptoms have been found in 38% of non-hospitalised patients as long as 23 months after onset [10], with fatigue, breathlessness, difficulty concentrating, memory problems and pain being the most common reported symptoms [5,11,12,13,14] 

Significant fluctuations in symptoms have been demonstrated by assessing the same patients at different time points in their journey [15], with up to 93% of patients reporting symptoms fluctuating over time [7]. Symptoms of fatigue and breathlessness were found to increase in one group of patients from 5 months after LC onset to 12 months [11], with other symptoms such as hair loss decreasing. This relapsing nature of LC has been further observed with symptoms increasing at 30 days post onset, decreasing at 60 days and then increasing again after 90 days [16]. This suggests that the unknown trajectory of this condition may be difficult to predict and that patients may need to access services as and when their symptoms deteriorate. 

The Leeds Long COVID Community Rehabilitation Service (LLCRS) was developed in response to NHS England’s commissioning guidance (2023) for a broad-based multi-disciplinary team to provide holistic, symptom-led rehabilitation for those whose symptoms were having a significant impact on their daily activities. In particular, with the largest number being people of working age, it aimed to support them in ways that enabled them to remain in work or return to work in a vocational rehabilitation programme. The multidisciplinary team in Leeds consists of occupational therapists, physiotherapists, dietitians, psychology professionals, rehabilitation assistants, a GP with a special interest and consultants in medical specialities of rehabilitation medicine, respiratory medicine and cardiology [17].

The debilitating nature of LC, with its effects on so many organ systems and its fluctuating and protracted nature in some, is well known. However, we lack knowledge of the long-term health effects of this novel condition. We wanted to know whether those being discharged from our service fully recover to their pre-COVID-19 status or whether they continue to have lingering symptoms. The aim of this study was to evaluate the long-term health outcomes in a sample of patients discharged from the service. We wanted to know the status of their LC symptoms, whether they are accessing any other services, what support they continue to require and how LC has impacted their ability to live, work and interact within society.

## 2. Materials and Methods

This service evaluation study was approved locally by the Leeds Community Healthcare NHS Trust. The Health Authority (HRA) toolkit was completed and confirmed as not needing any further national HRA ethical approvals to undertake this service evaluation. A standard follow-up questionnaire with the usual service Patient Reported Outcome Measures (PROMs) was sent to patients who had been discharged from the service and had consented to be contacted for service evaluation purposes. 

### 2.1. Participant Identification

Participants were identified by using an electronic patient record database for all patients who had been managed for LC in the LLCRS and discharged for more than 3 months. Inclusion criteria for the service evaluation were confirmed diagnosis of LC (as per NICE guidelines) [1], consented to service evaluation and research, initial EQ-5D-5L completed and documented in record, and evidence of ‘engagement’ with the service. ‘Engagement’ was set as a minimum of at least one consultation with a therapist documented on their clinical record. These criteria were applied to the list of discharged patients and postal questionnaires sent, along with information about the service evaluation study. Participation was voluntary and participants were free to withdraw at any point without having to give a reason for withdrawal. Deceased patients and those without adequate information were excluded from the study. 

The questionnaires included were the standard PROMs used in the service, i.e., Modified C19-YRS, EQ-5D-5L, myalgia encephalomyelitis/chronic fatigue syndrome (ME/CFS) screening and a qualitative questionnaire to capture the patient’s perception of their health state. These were agreed by the LLCRS Patient Carer and Public Involvement (PCPI) group. 

### 2.2. Patient Reported Outcome Measures (PROMs)

#### 2.2.1. C19-YRS

The COVID-19 Yorkshire Rehabilitation Scale (C19-YRS) was the literature’s first condition-specific PROM developed to measure the symptoms, functioning and disability associated with COVID-19. The C19-YRSm is a modified version of the original C19-YRS with 17 items and four sub-scales. Each item has a 4-point response category: 0, no problem to 3, severe problem [18]. The subscales (range) are Symptom Severity (0–30), Functional Disability (0–15), Other Symptoms (0–25) and Overall Health (0–10). The evaluation of psychometric properties of the C19-YRSm revealed it is a valid, reliable and responsive measure [19]. The Minimal Clinical Important Difference (MCID) has been estimated to be 4 points for the Symptom Severity subscale and 4 points for the Functional Disability subscales. 

#### 2.2.2. EQ-5D-5L

The EuroQol EQ-5D-5L is a health-related quality-of-life measure with five domains: Mobility, Usual Activities, Selfcare, Pain/Discomfort and Anxiety/Depression. Each item has five response categories ranging from 1 (no problems) to 5 (severe problems). Responses to each item are collated into a profile score which is converted into a health utility or index score using a country-specific algorithm (tariff or value set). The utility score reflects societal preference for health state and is measured on a metric from 0 (dead) to 1 (perfect health). The EQ-5D-5L scores are mapped onto the EQ-5D-3L (an alternative version of the instrument with 3 response categories advocated by the National Institute for Health and Care Excellence, NICE) using a standard mapping crosswalk algorithm to derive UK utility values [20]. 

### 2.3. NICE Criteria for ME/CFS 

All of these symptoms should be present to meet the NICE criteria for ME/CFS [21]:

- Debilitating fatigue that is worsened by activity, is not caused by excessive cognitive, physical, emotional or social exertion, and is not significantly relieved by rest.
- Post-exertional malaise after activity in which the worsening of symptoms:
  is often delayed in onset by hours or days
  is disproportionate to the activity
  has a prolonged recovery time that may last hours, days, weeks or longer.
- Unrefreshing sleep or sleep disturbance (or both), which may include:
  feeling exhausted, feeling flu-like and stiff on waking
  broken or shallow sleep, altered sleep pattern or hypersomnia.
- Cognitive difficulties (sometimes described as ‘brain fog’), which may include problems finding words or numbers, difficulty in speaking, slowed responsiveness, short-term memory problems, and difficulty concentrating or multitasking.

Patients were given the option of either completing the questionnaire and returning it by post or completing it with a clinician during a phone call. Patients had to re-confirm their participation in the service evaluation in the relevant section of the questionnaires.

## 3. Results

A total of 2124 patients had been discharged from LLCRS since its opening in September 2021. The inclusion criteria for this service evaluation study were met by 460 patients who were sent the agreed questionnaires. Completed questionnaires were returned by 118 patients. Six participants did not re-confirm their consent for the study and hence a final dataset of 112 participants was included in the analysis. Demographics for the participants are shown in Table 1. 

Participants had had LC for an average of 37.6 months and were 9.7 months post-discharge from LLCRS. There was a greater number of females in the sample (62%); participants were predominantly white (75%) and had an average age of 58.5 years. 

The average C19-YRSm and EQ-5D-5L scores at the different time points are shown in Table 2. 

Of the 112 respondents, only 11 participants reported that they had returned to pre-COVID-19 health (9.8%) with 90.2% of participants continuing to experience symptoms of LC (Table 3). However, 64.2% of these participants reported their symptoms were the same or had improved since discharge from LLCRS. Almost equal numbers of participants were in employment at follow-up, with no changes to their role since their COVID-19 illness (45%). Of those who had experienced some sort of change to their job or role (43%), the largest group was the 20% of participants who had either had to retire or change their job. 

There was a total of 52 new referrals to NHS services reported after discharge from LLCRS reported by participants, excluding visits to general practitioners. 

The private and NHS services accessed by participants after discharge from LLCRS (Table 4) include GP, physiotherapy, psychology, pain management, exercise class, neurology, respiratory, rheumatology, hearing services, reflexology, massage therapist, cardiology, gastroenterology, ENT, dietitian, orthopaedics, meditation, yoga, hypnotherapy, Nuffield Long COVID programme, personal trainer, peer support group, CUCs, shared harmonies (online singing course for breathing control), haematology, hospital admission, access to work, endocrinology, asthma nurse, hepatology, chiropractor and acupuncture. 

A total of 48 patients (43% of the sample) met the NICE diagnostic criteria for suspected ME/CFS (Table 5).

## 4. Discussion

The principal findings of this service evaluation study are that 90.2% patients who have been discharged from the LLCRS (and participated in this study) are still exhibiting significant symptom burden and functional disability from LC (at 3 years post-infection) and have not recovered to their pre-COVID-19 heath state. A very small proportion of patients who have engaged with the LLCRS made a full recovery to their previous heath state prior to COVID-19 infection (9.8%). The mean EQ-5D-5L index value at post-discharge follow-up was 0.53, which is comparable to the mean EQ-5D-5L index scores of several other LTCs such as COPD, heart failure and multiple sclerosis (see Table 6).

We believe that this is one of the first studies to measure outcomes of LC patients at different time points along their journey after being referred to a specialist LC clinic, specifically at discharge and 3 years post discharge. One study reported mean EQ-5D-5L index scores of 0.54 in patients referred to an NHS community LC clinic at assessment [13]. Our mean EQ-5D-5L index score of 0.52 at entry to the service compares well to these findings, suggesting a similar population. However, we are unable to make comparisons in terms of improvement whilst in the service as no other study has assessed outcomes at discharge or beyond. Other studies amongst the general population self-reporting LC have found average EQ-5D-5L index scores of 0.49 [14] and 0.75 [22] but these may include a range of respondents and will not be made up entirely of patients referred for rehabilitation. 

There is a general trend in the literature of reported recovery from LC within the general population over time [12,23]. One study reported that only 6% of patients with mild to moderate COVID-19 still had symptoms at 24 months [5] whilst another found 17.2% had not fully recovered at 24 months [24]. Furthermore, another study reported that average EQ-5D-5L index scores recovered (mean 0.84) to pre-COVID-19 levels (mean 0.82) amongst a group of 300 non-hospitalised patients, 20% of them with self-reported LC, over a 2-year period [25]. This cross-sectional retrospective study included anyone with previous COVID-19 and was not selective to those with LC like our study. Our sample is likely be biased towards those with persistent symptoms and thus these will be overrepresented, but it does represent a specific group of patients for whom this is likely to be a long-term condition.

Findings in this study add weight to this emerging picture of Persistent LC (PLC) as a long-term condition with clear evidence that even after being treated in a specialist LC service the majority of patients continue to face overwhelming and debilitating symptoms for as long as 3 years. 

**Table 6:** Comparison of EQ5D-5L Index Scores in this population with LC and other chronic long-term conditions [26,27,28,29,30].

Condition	EQ-5D Index (SD)
Healthy population	0.92 (0.17)
COPD	0.68 (0.24)
Heart failure	0.60 (0.25)
Multiple sclerosis	0.59 (0.29)
Long COVID (this service evaluation study)	0.53 (0.29)

PROMs assessed during participants’ time with LLCRS all show improvements from initial assessment to the point of discharge. Using a minimally clinical important difference (MCID) of 4 [19], symptom severity was shown to improve significantly from a mean of 18.4 at initial assessment to 13 at discharge. Mean Functional Disability scores decreased during a participant’s time within the LLCRS from 6.7 to 5.9 but this does not reach clinical significance. Overall health scores also improved from a mean of 4.9 to 6 at discharge but this does not quite return to the pre-COVID-19 average of 7.3. 

EQ-5D-5L index scores improved, indicating a significant change in quality of life from entering the service (0.52) to discharge (0.65) by a mean EQ-5D-5L index score of 0.13, which is above the smallest clinically meaningful change of 0.08 [31]. Furthermore, there was a clinically significant improvement in the EQ-5D-5L VAS mean score of 13.1 (MCID value 7.5 [32]) between initial assessment and discharge from the service. This demonstrates that targeted interventions used within the LLCRS were effective in reducing symptom burden and improving the functional levels of individuals who engaged with clinicians. However, the mean EQ-5D-5L index value of 0.65 at discharge is still well below that of the healthy population at 0.92. This suggests the service is getting patients to a level of self-management and stability in the condition rather than full recovery. This also reflects on the nature of this novel condition.

Outcomes are seen to deteriorate after discharge in both the EQ-5D-5L and the C19-YRSm with average symptom severity increasing significantly from 13 to 15.1 after discharge [19]. Average EQ-5D-5L index scores after discharge dropped below initial EQ-5D-5L index scores by 0.12, representing a significant deterioration in functional levels. Furthermore, EQ-5D-5L VAS scores deteriorated between discharge and follow-up, reducing by 7.3 (from 65 to 57.7), but not quite reaching the clinical significance of 7.5, indicating that participants are experiencing a substantial drop in functional ability once they have been discharged. This highlights the value of the service in stabilising the long-term condition and the need for the service to continue to provide regular input to avoid any such deterioration in health state. This is also reflected in the fact that there were significant referrals to other services after discharge to manage the flare-ups of LC which we know is a fluctuating condition. 

The employment status in this cohort was adversely affected in 43% of patients. Other national studies have reported that this change can be as much as 62% [6] and indeed findings from local data show that in 75% of patients at entrance to the LLCRS there had been a negative change to their working situation. It may be that as we follow participants further down the line, some who were off sick in the initial weeks and months of their diagnosis will be able to return to work and indeed some who had their hours temporarily reduced are able to return to their usual hours with the support and guidance from the specialists within LLCRS. This highlights the crucial role that specialist services for long COVID play in vocational rehabilitation and supporting reasonable adjustments within the workplace to enable as many people as possible to remain in their roles or to return to work with appropriate support. Identifying common themes in specific tasks or roles which represent the biggest challenges for people with LC symptoms would be incredibly useful for employers and service providers and would warrant a specific piece of work for further development. 

The magnitude of the symptom burden experienced by participants at follow-up is clearly illustrated in the very low symptom burden and Functional Disability score at pre-COVID-19 assessment compared with an average symptom severity of 15 at follow-up and with 40% reporting more than three other significant health problems. Fit and well individuals have now developed further health conditions as a result of their ongoing LC symptoms. This reiterates a new-onset long-term condition of LC in most of these individuals with a significant burden to the healthcare system (as evidenced by healthcare services referrals they have had since discharge) and economy.

As many as 43% of participants with ongoing LC symptoms continue to experience symptoms which may fit with a suspected ME/CFS diagnosis and would warrant further assessment, reporting significant ongoing symptoms for more than 3 months of debilitating fatigue, post exertional malaise, unrefreshed sleep and cognitive difficulties as described by the National Institute for Health and Care Excellence (NICE) (2021) [21]. This is generally in keeping with the high prevalence of ME/CFS within LC populations in the literature with studies reporting 45.2–58% of LC patients fitting these criteria [33,34].

The NICE guidelines criteria for ME/CFS (2021) [21] were used in this study; however, we found the wording of the first question confusing and unclear to participants and it was discovered that although many participants had fatigue scored on other PROMs they had not selected the first item on debilitating fatigue in the NICE ME/CFS criteria. Whilst recent studies have used the Institute of Medicine (IOM) criteria or the Canadian Consensus Criteria (CCC) to identify the prevalence of ME/CFS in those with LC, we used the UK NICE Guidelines criteria as these are more widely used within our local services [35,36]. We were also mindful of the time taken to complete an additional long questionnaire, particularly in this population with fatigue and cognitive difficulties as their most frequent challenges. 

This study has several limitations. As this is a self-selected sample (24% response rate), it is likely that those patients who are still struggling with PLC have responded. However, this is similar to all questionnaire surveys and does highlight that there is a proportion of patients who have been worse since discharge and essentially have developed an LTC. The actual proportion of LC patients who end up with an LTC needs further prospective research. Studies such as LOCOMOTION are ideally placed to undertake such work on larger datasets. LOCOMOTION is an NIHR-funded UK multi-site study with mixed-method research and engagement of front-line clinicians and patients to co-design equitable services and develop training packages and resources for both [37]. Using the NICE criteria for ME/CFS was a limitation as discussed above and adding few other criteria such as those of the Institute of Medicine (IOM) or Centre for Disease Control and Prevention (CDC) would have added value to understanding the overlap between the conditions. This area needs further prospective research as it determines how the services for these overlapping conditions could consider integration (combined services) to provide a robust clinical and active research service that moves this area of medicine forward. 

## 5. Conclusions

From a clinical perspective, the learning from this study is that although 90.2% of the participants in this study had not returned to their pre-COVID-19 levels of health, 74% of the total sample population reported their symptoms were either the same or improved (including returned to pre-COVID-19 levels). This indicates that despite 43% of participants also fitting a potential clinical diagnosis of ME/CFS, the rehabilitation model employed by the LLCRS successfully supports people in managing their symptoms through the guidance of specialised, experienced clinicians regardless of their specific diagnosis. 

With a recent move towards reduction in specialist LC services with clinicians who have developed expertise in managing LC, this study highlights the huge gap between unmet need and resources being planned for this patient group. These patients need holistic multidisciplinary care including optimised medical management, therapy input, vocational rehabilitation and long-term continuous input given the long-term condition (LTC) nature of their illness. Otherwise, it is likely these patients will then present to services where there is little understanding of the complexities of LC and no experience of how to provide appropriate support and rehabilitation, leading to further decline in functional status, increased health needs and further burden on the NHS along with greater dependence on social care or productivity loss. 

## Figures and Tables

**Table 1:** Sociodemographic characteristics for all participants included in the study.

Demographics	Number	%
**Total Number of Responses**	112 58.5 (22-81)	
Median age (range)		
**Sex** Male	42	37.5
Female	70	62.5
**Ethnicity**		
White	84	75
Mixed	0	
Asian or Asian British	2	1.8
Black or Black British	1	0.9
Other ethnic groups	0	
Unknown	25	22.3
**Employment**		
Employed—no change	50	45
Had to retire/change jobs	22	20
Reduced working hours	15	13.4
Changes to job/role	7	6.3
Missing/unknown	7	6.3
Lost job	3	2.7
Disability	3	2.7
Unemployed	3	2.7
Sick Leave	1	0.9
**Body Mass Index**		
Median (range)	28.7	(14.8–47.4)
Underweight	1	1
Healthy weight	19	17
Overweight	32	29
Obese	36	32
Unknown	24	21
**Co-morbidities (>3)** significant co-morbidities)	40	35.7
**Duration of LC**—Median (range)	37.06 months (15.08–49.51) 9.7 months (3.9–26.5)	
**Time since discharge from the service**—Median (range)		

**Table 2:** PROM values at different time points of the service evaluation.

PROM (SD)	Pre-COVID-19 Mean (SD)	Initial Mean (SD)	Interim Mean (SD)	At Discharge Mean (SD)	Post-Discharge Mean (SD)
C19—YRSm SS	**4**	**18.4**	**17.3**	**13**	**15.1**
	(4.59)	(4.94)	(5.31)	(7.54)	(6.06)
C19-YRSm FD	**1.2**	**6.7**	**6.2**	**6**	**5.9**
	(2.47)	(3.32)	(2.65)	(3.6)	(4.48)
C19-YRSm OH	**7.3**	**4.9**	**5.5**	**6**	**5.2**
	(2.76)	(1.77)	(1.98)	(2.03)	(2.21)
EQ-5D-5L Index Value	-	**0.52**	**0.52**	**0.65**	**0.53**
	-	(0.27)	(0.25)	(0.23)	(0.29)
EQ-5D-5L VAS	-	**51.9**	**53.11**	**65**	**57.67**
	-	(20.84)	(16.51)	(20.46)	(20.19)

SD—Standard Deviation, C19-YRSm—COVID-19 Yorkshire Rehabilitation Scale-Modified, SS—Symptom Severity, FD—Functional Deficit, OH—Overall Health, VAS—Visual Analogue Scale.

**Table 3:** Reported changes since discharge from LLCRS.

Change Since Discharge from LLCRS	No. of Patients (*n* = 112)	%
Back to pre-COVID-19 health	11	9.8
Greatly improved	16	14.2
Some improvement	31	28
Same as when discharged	25	22
Somewhat worse	21	19
A lot worse	7	6.2
Unanswered	1	0.8

**Table 4:** Other health services accessed since discharge from LLCRS.

	Number of Patients (*n* = 112)	%
Participants accessing any other health service since discharge	48	43
**Participants with at least 1 NHS referral for LC management since discharge** **(excluding GP)**	**31**	**28**
1 NHS referral since dc	16	14.2
2 NHS referrals since dc	13	12
4 NHS referrals since dc	1	0.9
6 NHS referrals since dc	1	0.9

**Table 5:** Patients who met the criteria for a suspected diagnosis of ME/CFS.

ME/CFS Screening Number of Criteria Items Identified as Met by Patients	Number of Patients (Total *n* = 112)	%
4 items	48	43
3 items	13	11.6
2 items	18	16
1 item	14	12.5
0 items	12	10.7
Did not answer	7	6.2

## Data Availability

Anonymised data can be obtained by contacting the corresponding author.
